# Is the future of general practice safe for patients?

**DOI:** 10.3399/bjgp19X702629

**Published:** 2019-05

**Authors:** Ian Bennett-Britton, Chris Salisbury

**Affiliations:** Centre for Academic Primary Care, Bristol Medical School, University of Bristol, Bristol.; Centre for Academic Primary Care, Bristol Medical School, University of Bristol, Bristol.

## INTRODUCTION

General practice is changing rapidly, driven by policy demands for new models of care to address an expanding, ageing, and increasingly medically complex population.^1^ Such change presents opportunities to improve all aspects of care; however, questions remain about the risks to patient safety. These risks can be grouped into: those related to changes in workforce and workload; those related to changes in infrastructure and models of care; and those related to limitations of existing mandatory patient safety systems in general practice.

## WORKFORCE AND WORKLOAD

The General Practice Forward View^1^ (GPFV) set out NHS England’s strategy for general practice up to 2020, committing to 5000 additional doctors and a minimum of 5000 other staff, including mental health therapists, clinical pharmacists, and physician associates (PAs). The roles of new and existing staff are expanding to more efficiently use GP capacity. Although investment in general practice is welcomed, there is uncertainty regarding safe limits of delegation and supervision of staff in existing and new roles.

Some of the risks surrounding the workforce changes are illustrated by the GPFV’s commitment to increasing PA numbers, from 31 known to be working in general practice in 2016, to 1000 by 2020.^2^ PAs are presently regulated on a voluntary basis despite commitments to rapid expansion of the role since 2016. Fortunately, in October 2018, the Department of Health committed to developing statutory regulation for PAs.^3^ However, the value of this will be defined by its terms. The clinical governance arrangements under which PAs may work remain ill defined, despite warnings that these are of *‘critical importance in ensuring the quality and safety of their work’*.^4^ Such uncertainty risks inappropriate utilisation of staff in stretched general practices.

## INFRASTRUCTURE AND MODELS OF CARE

Among other drivers, advances in technology are transforming general practice infrastructure and models of care, enabling the growth of a range of services from artificial intelligence-facilitated patient triage to video-call consultations.^5^ Such developments are compelling; however, concerns remain that interventions are being applied to patients without adequate evidence of safety.^5^

Examples of this are summarised in the Care Quality Commission’s (CQC) report on independent online primary health services.^6^ Initial inspections found that 30 of 35 providers did not fully meet criteria consistent with safe care,^6^ with failures across prescribing, safeguarding, patient identification, and information sharing. Such failures highlight the risk of harm in an environment where, by the CQC’s own admission, *‘the pace of advancement in technology has outpaced the evolution of the regulations’*.^6^

## LIMITATIONS OF EXISTING MANDATORY PATIENT SAFETY SYSTEMS

The absence of clear limits of task delegation and supervision of new and existing staff, and evidence of failures in the provision of online medical services, illustrate the risks presented by the transformation of general practice. The lack of consistent evaluation to identify and mitigate such risks, coupled with the pace and disparate nature of such changes, leads one to question what systems are already integrated into general practice that would highlight when patient safety is at risk.

The following section provides an overview of the mandatory ‘safety-net’ systems in general practice today, categorising by those at the organisational, clinician, and patient level.

### Organisational level

The principal mechanism to ensure patient safety at the organisational level is CQC regulation. CQC assurance processes^7^ include data monitoring and targeted inspections to discern whether services are safe, effective, caring, responsive, and well led.

One of the key safety indicators described by the CQC is the propensity to report safety incidents and learn from them.^7^ Reporting of incidents resulting in severe harm is mandatory, but the CQC advises that all incidents, including near misses, should be reported to a national database, the National Reporting and Learning System (NRLS). NRLS data indicate that only 8383^8^ general practice incidents were reported to have occurred between October 2016 and September 2017, an average of one incident per GP practice annually. Variation in patient safety incident reporting was explored in an interview study of primary healthcare staff in London.^12^ Participants described inadequate time to engage in these activities, and *‘disincentives for responding to and acting on safety issues and concerns, with few reported benefits’*.^12^ Such evidence suggests incident-reporting systems are unlikely to consistently identify and ameliorate sources of patient risk.

### Clinician level

Professional regulation is the principal clinician-level ‘safety-net’ mechanism. It functions primarily through revalidation, which aims to ensure clinicians are *‘not just qualified, but safe’*.^9^

The extent to which revalidation of doctors improves patient safety is debated, with the principal evidence coming from the research of the UK Medical Revalidation Collaboration.^13^ Only 20% of surveyed doctors thought revalidation improved patient safety, the minority (23%) thought revalidation would identify failing doctors, and most (58%) *‘made no change to their clinical practice, professional behaviour, or learning activities, as a result of their most recent appraisal’*.^13^ As a consequence of this, and other assessments of the impact of revalidation,^9^ the GMC has committed to improvements.

A future of increasingly blurred professional boundaries^1^ requires that regulatory regimes of staff working in similar spheres are consistently detailed. However, the disparity in revalidation intensity between doctors and registered nurses,^10^ and only recent commitment to developing mandatory professional regulation of PAs,^3^ highlight an increasingly relevant inconsistency (Box 1).

**Box 1.** Comparison of current regulatory regime by clinician group

	**GPs^9^**	**Registered nurses^10^**	**Physician associates^11^**
**Regulation**	Mandatory: General Medical Council (GMC)	Mandatory: Nursing and Midwifery Council (NMC)	Presently voluntary:^a^ Faculty of PhysicianAssociates (FPA)
**Appraisal**	Mandatory: annually	Recommended: annually	Recommended: annually
**Revalidation frequency**	5-yearly	3-yearly	6-yearly for voluntary register
**Individual responsible for recommending revalidation**	Independently allocated: ‘responsible officer’^9^ (97%) or GMC-approved ‘suitable person’ (<1%).^9^ Otherwise for GMC assessment (2%)	Individual nurse choice: ‘confirmer’^10^ — normally line manager or NMC-registered individual. Otherwise, any regulated healthcare professional	FPA checks compliance for those who are voluntarily registered
**CPD (annual)**	50 hours^b^	11.7 hours^b^	50 hours for voluntary register
**Feedback requirements**	Once every 5 years: patient and colleague surveys	Once every 3 years: five pieces of patient or colleague feedback: ‘formal or informal; written or verbal’^10^	No requirement
**Additional revalidation requirements**	Reviews of: complaints and compliments significant events quality improvement activity; and reflective practice	Five written reflective accounts and discussion about these with someone registered with the NMC 450 hours of nursing practice	Recertification exam: 200 single best answer questions every 6 years. Exam not specific to specialty of practice

a *In October 2018 the Department of Health committed to developing mandatory regulation of physician associates*.^3^

b Mandatory average (mean) annual commitment. CPD = continuing professional development.

Uncertainty over the current ability of clinician-level safety mechanisms to ensure patient safety raises doubts over their suitability for monitoring future models of care.

### Patient level

Patient-level ‘safety-net’ mechanisms function through accountability and feedback to help identify areas of risk. These consist of surveys, written complaints, and online review systems.

As an overview, patient-level safety mechanisms are undermined by low levels of engagement, which, coupled with the asymmetry of information that defines the doctor–patient relationship, limit their usefulness for identifying risks to patient safety, now and in the context of the future of general practice.

## IS THE FUTURE OF GENERAL PRACTICE SAFE FOR PATIENTS?

Questions regarding the safety of future models of care draw attention to what is known about the safety of existing models of general practice. The presented overview of existing mandatory safety assurance systems highlights challenges that are likely to be exacerbated by the future of general practice. This underlines the need for research to consider alternative approaches to ensure the safety of existing and future models of care.

Alternative patient safety mechanisms may move away from the present regimes of infrequent, resource-intensive assessments of mostly self-collated evidence — often with perverse-incentive structures — towards independent and continuous assessments based on triangulation of a wide range of variables. In the context of increasingly blurred professional boundaries, more sophisticated assessment regimes may place greater emphasis on situational competence and appropriate supervision, rather than professional status. The development of such safety systems may be facilitated by strategic moves towards larger organisational general practice units^1^ and advances in machine learning technology.

The future of general practice is reliant on the ability of services to continuously evolve to respond to new challenges. The safety of this future depends on corresponding investment and innovation in patient safety assurance mechanisms, and, crucially, incentive structures that support meaningful and consistent engagement with them.
